# Robust Functionalization of Large Microelectrode Arrays by Using Pulsed Potentiostatic Deposition

**DOI:** 10.3390/s17010022

**Published:** 2016-12-23

**Authors:** Joerg Rothe, Olivier Frey, Rajtarun Madangopal, Jenna Rickus, Andreas Hierlemann

**Affiliations:** 1 ETH Zurich, Department of Biosystems Science and Engineering, Bio Engineering Laboratory, Mattenstrasse 26, CH-4058 Basel, Switzerland; olivier.frey@insphero.com (O.F.); andreas.hierlemann@bsse.ethz.ch (A.H.); 2 Agricultural and Biological Engineering, Biomedical Engineering, Physiological Sensing Facility at the Bindley Bioscience Center and Birck Nanotechnology Center, Purdue University, West Lafayette, IN 47907, USA; rajtarun.madangopal@nih.gov (R.M.); rickus@purdue.edu (J.R.); 3 Intramural Research Program of the National Institute on Drug Abuse, National Institutes of Health, Baltimore, MD 21224, USA

**Keywords:** electrodeposition, microelectrode array, pulse potential waveform, voltage pulses, pulsed potentiostatic deposition, complementary metal-oxide-semiconductor (CMOS), platinum, gold, poly(phenylenediamine) PPD, poly(ethylenedioxythiophene) PEDOT

## Abstract

Surface modification of microelectrodes is a central step in the development of microsensors and microsensor arrays. Here, we present an electrodeposition scheme based on voltage pulses. Key features of this method are uniformity in the deposited electrode coatings, flexibility in the overall deposition area, i.e., the sizes and number of the electrodes to be coated, and precise control of the surface texture. Deposition and characterization of four different materials are demonstrated, including layers of high-surface-area platinum, gold, conducting polymer poly(ethylenedioxythiophene), also known as PEDOT, and the non-conducting polymer poly(phenylenediamine), also known as PPD. The depositions were conducted using a fully integrated complementary metal-oxide-semiconductor (CMOS) chip with an array of 1024 microelectrodes. The pulsed potentiostatic deposition scheme is particularly suitable for functionalization of individual electrodes or electrode subsets of large integrated microelectrode arrays: the required deposition waveforms are readily available in an integrated system, the same deposition parameters can be used to functionalize the surface of either single electrodes or large arrays of thousands of electrodes, and the deposition method proved to be robust and reproducible for all materials tested.

## 1. Introduction

Microelectrode arrays (MEAs) constitute a widely used platform that enables spatially highly resolved parallel sensing [1,2,3,4,5,6]. Owing to the advances in microfabrication technology, established methods for manufacturing microelectrodes from a large variety of materials over a wide range of geometries and spatial arrangements are available [7,8,9,10]. In most cases, arrays of electrodes are fabricated on the same substrate using the same materials due to the parallel and planar nature of microfabrication techniques. Desired surface properties of microelectrodes include: (I) large active surface area and/or low impedance for effective charge-transfer, i.e., high signal-to-noise ratio during recordings and high stimulation efficacy [11,12]; (II) good adhesion or chemical binding properties for biochemical recognition units or matrices that encage biomolecules such as enzymes [13,14], or function as a size discrimination or anti-interference layers to reduce cross-sensitivity [15]; and (III) robustness against electrode fouling [16]. Many of these properties can be obtained by specific surface functionalization of the electrodes with the aim of attaining biosensor systems that are capable of performing parallel measurements through many electrodes or that enable measuring different analytes at the same time. Multi-analyte sensing with the same system requires that various subsets of electrodes on an array can be functionalized with different layers.

Micrometer dimensions of the electrodes and electrode distances (<100 µm) obviate the application of manual coating methods, such as depositing drops of coating solutions onto microelectrodes. Also, dipping methods do not enable selective and local deposition on planar electrode arrangements and arrays. Good spatial control of the deposition process and high reproducibility of the quality and morphology of coating over several sensor electrodes are prerequisites to fabricate electrode arrays with different specific sensing properties and to obtain low sensor–signal crosstalk. This holds particularly true for large arrays of densely packed microelectrodes.

Electrodepostion-based surface modification techniques satisfy several of the criteria listed above, as the deposition process relies on a chemical reaction coupled with a charge transfer through the connected electrode(s). Traditional electrodeposition schemes rely on galvanostatic methods, in which constant currents are applied to obtain a constant deposition rate. To improve the quality of the deposited layers, several protocols for applying pulsed currents instead of a constant current have been established [17]. The current has then to be adjusted with respect to the deposition area in order to obtain the desired current density. In most cases, however, the exact area of the target electrode may be unknown and to find a robust deposition protocol for the respective functional layer is challenging. This challenge holds particularly true if the deposition should be done on electrodes of different size. The deposition protocol needs to be adapted for every coating material and coating scenario. Pulsed potentiostatic deposition, in contrast, relies on voltage pulses, which allows for applying the same parameters independent of the overall electrode area. Publications reporting on the usage of voltage pulses for electrodeposition can be found for applications that are largely different from those described in this paper. Horkans and Romankiw reported on pulsed potentiostatic deposition on a large gold electrode [18]. Plyasova et al. performed electrostatic depositions of platinum on gold and glassy carbon substrates, showing differences in the nanostructure of the surface upon varying the deposition potential [19]. Using square-wave potential pulses, Liu et al. 2014 grew nanodendrites with a different morphology by altering the anodic potential [20].

A robust deposition protocol and method is particularly important for large microelectrode arrays, in which electrode size and the number and arrangement of electrodes to be functionalized can vary. Further, for developing a protocol for a new material or a new electrode design, it would be highly desirable that deposition parameters be tuned and determined using a single electrode and subsequently be applied to a larger subset of the microelectrode array without modification.

In this paper we present a universal electrodeposition method that relies on voltage pulses rather than on the more commonly used current pulses to achieve spatially confined growth of both metallic and polymer layers on electrode surfaces. The voltage-pulse-based deposition method presented in this paper enables the use of the same parameters for different electrode sizes (diameters of 5–50 μm). Moreover, this method is highly scalable in that it enables deposition on different and large numbers of electrodes in parallel. We demonstrate the deposition method for electrodeposition of two key classes of materials, (i) the metals Pt black and gold, and (ii) the polymers poly(phenylenediamine), also known as PPD, and poly(ethylenedioxythiophene), also known as PEDOT. Deposition is carried out on an array of 32 × 32 platinum microelectrodes, placed on a fully developed complementary metal-oxide-semiconductor (CMOS) microelectronic chip. Adjustment of the material-specific deposition parameters allows for controlling the roughness of the surface, the layer morphology, and the capacitive properties or permselectivity of the deposited films.

## 2. Materials and Methods

### 2.1. CMOS Microelectrode Array

The electrodeposition and functionalization protocol was developed on a fully integrated CMOS potentiostat chip [21] featuring an integrated 32 × 32 array of platinum electrodes (cf. Supplementary Figure S1a). The system formed part of a standard three-electrode setup including an on-chip platinum counter electrode and an external Ag/AgCl/KCl (3 M) reference electrode. The platinum working electrodes were disk electrodes with a standard diameter of 25 μm or featuring different diameters ranging from 5 to 50 μm. The electrodes were arranged on a 32 × 32 grid configuration at 100 µm pitch. The platinum layer (270 nm) for the microelectrodes was deposited and structured through ion-beam deposition and ion-beam etching processes. A passivation stack (1.6 µm), consisting of alternating SiO_2_ and Si_3_N_4_ layers was deposited on the chips and was structured by reactive ion etching to open the active areas of the electrodes. As a result, the platinum surface of the microelectrodes was located in a shallow recess (approx. 1.5-µm deep) of the passivation stack. 

The electronic circuits on the chip allowed for generation of arbitrary signal waveforms at a resolution of 2.8 mV. The voltages were generated on chip, and the measured currents were digitized on chip as well. The chip was connected to a field programmable gate array (FPGA, Xilinx Spartan 6), for signal processing. The experiments were controlled, and the data recorded on a computer through a USB connection. To perform experiments, a reservoir holding approximately 5 mL liquid volume was placed atop the electrode array (cf. Supplementary Figure S1b). The reservoir could be closed by means of a lid with holes for inserting the reference electrode and a nitrogen gas supply line.

### 2.2. Electrodeposition Protocol

Before usage, the microelectrodes were electrochemically treated by varying the electrode potential between –0.2 and +1.2 V vs. Ag/AgCl for 32 cycles in a de-aerated 0.5 M H_2_SO_4_ solution at a rate of 100 mV/s. The chips were then rinsed with deionized water (DI water) and blown dry with a nitrogen gun. 

**Figure 1:**
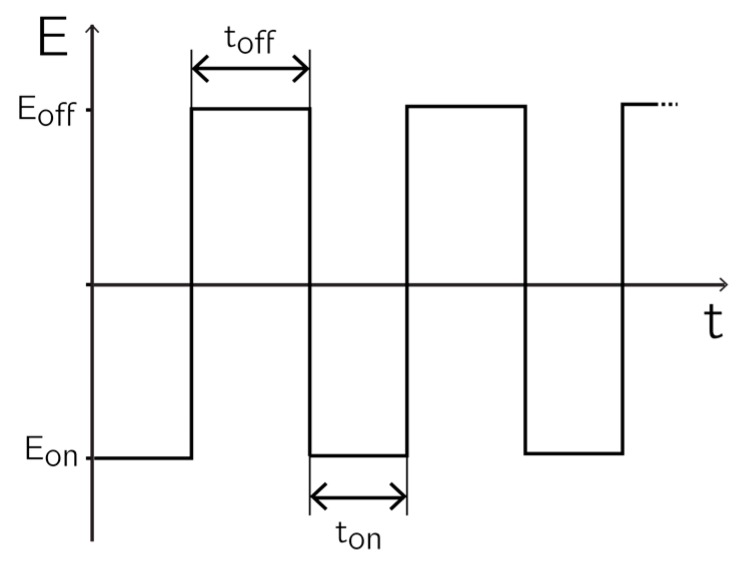
Voltage-pulse-based deposition scheme. Pulse shape for cathodic deposition of, e.g., Au or Pt black. During t_on_, the potential E_on_ is applied at the selected deposition sites; during t_off_, the electrodes are brought back to the initial open-circuit potential E_off_. For oxidative deposition reactions, the polarities have to be inverted.

Before deposition, the open-circuit potential (OCP) was determined with the help of the Ag/AgCl reference electrode and a platinum wire. The deposition scheme was performed by applying alternating potential pulses (see Figure 1). All depositions were performed by using 1 mL of deposition solution. The solutions were de-aerated by nitrogen bubbling for 5 min before deposition started. A plastic pipette tip mounted to a sonicating electric toothbrush was used for agitating the solution and for reducing diffusion limitations.

For deposition, a pulse of length t_on_ and potential E_on_ was applied to the working electrodes with respect to the reference electrode. E_on_ is the potential at which the deposition occurred. During t_off_, the electrode was brought back to the OCP by applying E_off_, so that the ionic species to be deposited was replenished in the diffusion layer. The relaxation time t_off_ was defined as the time after which no significant current could be measured anymore. 

The parameter windows for the different materials are listed in Table 1. Metals like platinum or gold could be electrodeposited by reduction of their salts in aqueous solutions [22]. In these cases, E_on_ was lower than E_off_ and the OCP. Compounds like phenol and phenylenediamine (PPD) polymerize upon electrochemical oxidation of the aromatic amine portion of the complex [13,23]. In that case E_on_ was larger than E_off_.

### 2.3. Electrochemical Characterizations

Electrochemical characterizations using cyclic voltammograms (CVs) were performed in de-aerated 0.5 M H_2_SO_4_ at a sweep rate of 100 mV/s, unless otherwise noted. A continuous nitrogen stream was maintained over the sample solution during the measurements.

### 2.4. Pt Black Deposition

A solution of hexachloroplatinic acid (17.5 mM) and lead(II) acetate trihydrate (0.03 mM) (from Sigma-Aldrich, Buchs, Switzerland) dissolved in DI water was used for electrodeposition of Pt black. Platinum black was deposited by using the parameters listed in Table 1. The pulse-widths t_on_ were varied between 0.1 and 0.8 s, t_off_ was set to 0.4 s in all cases. The total t_on_ times (t_on_* number of pulses) were kept constant for each set of pulse-widths by adapting the number of pulses. The depositions with different parameter sets were performed sequentially without renewal of the solution. 

The roughness factors were determined coulometrically from the hydrogen desorption region of the CVs measured in H_2_SO_4_ solution at a sweep rate of 100 mV/s [24].

### 2.5. Gold Deposition

Neutronex 309 solution (Enthone Inc., West Haven, CT, USA) was used for electrodeposition as provided. Two different parameter sets were selected (see Table 1), which allowed for the production of gold surfaces with different surface morphologies. 

### 2.6. Deposition of Conducting Polymers

PEDOT deposition solution was prepared by adding 20 mM 3,4-ethylenedioxythiophene (EDOT, 97%, Sigma Aldrich, Buchs, Switzerland) to a 1 wt % aqueous solution of poly(sodium 4-styrenesulfonate), PSS, MW ~70,000 g/mol, Sigma Aldrich, Buchs, Switzerland.

### 2.7. Deposition of Non-Conducting Polymers

For the PPD layer, o-phenylenediamine (100 mM, Sigma Aldrich, Buchs, Switzerland) was dissolved in de-aerated phosphate-buffered saline (PBS, Sigma Aldrich, Buchs, Switzerland). Amperometric calibration series for dopamine and ascorbic acid (both from Sigma Aldrich) were performed at 650 mV vs. an Ag/AgCl reference electrode in PBS.

## 3. Results

### 3.1. Pt Black Deposition

A layer of amorphous platinum black on top of the bare platinum electrodes increases the effective surface area while preserving a small geometric sensor area. The increase in effective surface area lowers the impedance of the electrode and allows for better charge transfer, which is beneficial for both sensing and stimulation scenarios [12].

**Figure 2:**
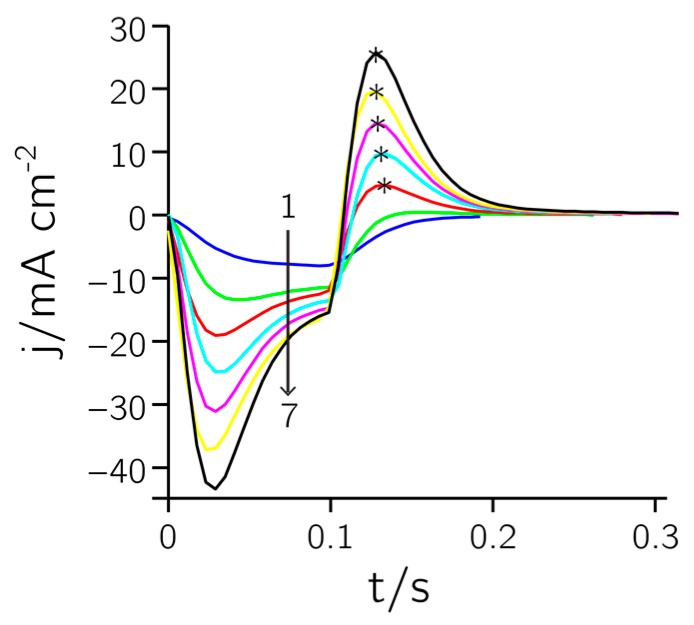
Deposition of Pt black. The curves marked 1 to 7 show the current current density versus time response of every 13th pulse as recorded during the deposition procedure (t_on_ = 0.1 s, E_on_ = 0 V, t_off_ = 0.4 s, E_off_ = 0.68 V, 160 cycles). The asterisks indicate the current density peak values for charging the double layer capacitance.

Figure 2 shows the current response during a deposition of Pt black (t_on_ = 0.1 s, E_on_ = 0 V, t_off_ = 0.4 s, E_off_ = 0.68 V, 160 cycles). Single pulses (every 13th pulse) were extracted at several time points and overlaid (1–7). The first negative section of the pulse is a combination of non-faradaic (i.e., charging of the double layer) and faradaic (i.e., metal deposition) currents. The second (positive) part of the pulse is only due to non-faradaic charging of the double layer. Both pulses increase over time, as the active surface area is becoming larger, which entails an increase of the double layer capacitance.

**Figure 3:**
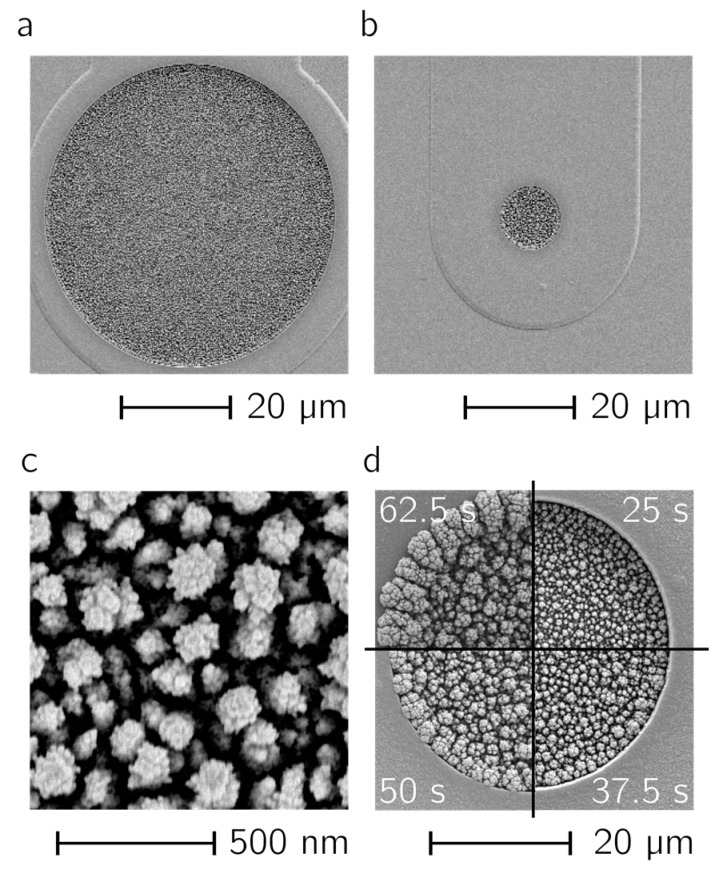
Deposited Pt black. SEM micrographs of deposited Pt black layers. The same deposition parameters have been used for a 50-µm (**a**) and a 10-µm-diameter (**b**) electrode; (**c**) Close up of the Pt black morphology and structure; (**d**) Different total t_on_ times, clockwise from top right: 25 s, 37.5 s, 50 s, 62.5 s for deposition on a 10-µm-diameter electrode.

In Figure 3a,b two electrodes with diameters of 50 µm (a) and 10 µm (b) are shown. The Pt-black layers were deposited by using the deposition parameters mentioned above. In both cases, the electrode surface is homogeneously covered, no overgrowth of the layers can be seen, and the layers show the same morphology upon optical inspection. The dendritic structure of the Pt black surface, which is the reason for the increase in active electrode area, is shown in Figure 3c. The effect of increasing the number of pulses can be seen in Figure 3d. Pt black was deposited in the recessed electrode area during a total t_on_ time of 25 s, 37.5 s, 50 s and 62.5 s (from top right clockwise); at 50 s the layer started to overgrow the recessed electrode opening. 

**Figure 4:**
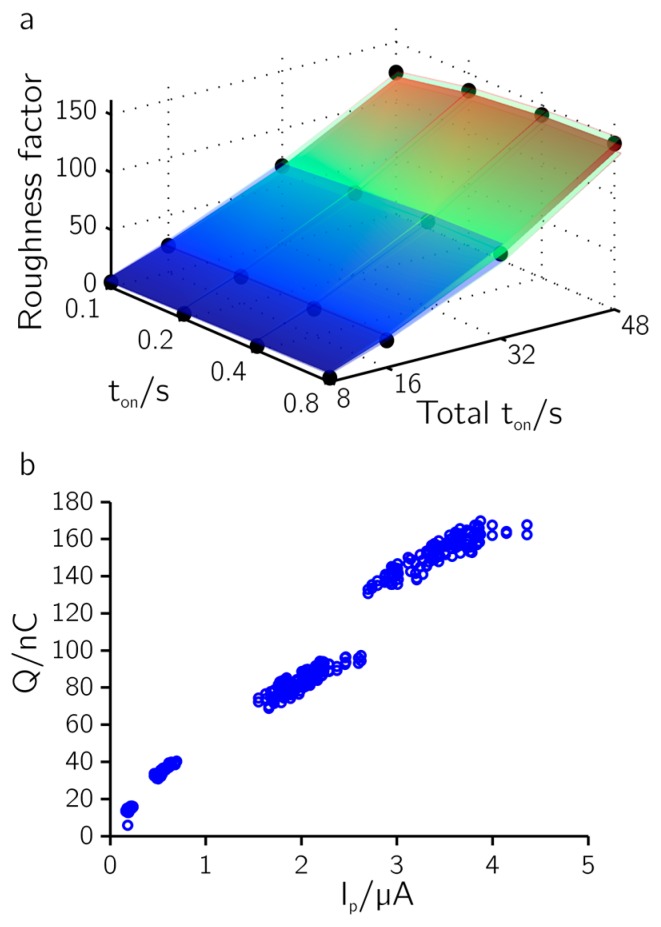
Pt black characteristics. (**a**) Roughness factor determined from integrating cyclic voltammograms (CVs) in H_2_SO_4_ from all electrodes of one chip for different deposition parameters (*n* = 64 electrodes per data point). Standard deviations are represented by semitransparent layers. (**b**) Integrated current from the hydrogen desorption region vs. maximum positive pulse height of the pulse train during deposition.

We further determined the roughness factors from the hydrogen desorption region of cyclic voltammograms (CVs) measured in a 0.5 M H_2_SO_4_ solution at a sweep rate of 100 mV/s [24] (Supplementary Figure S2). Figure 4a shows the results of measurements from all 1024 electrodes on a single chip. It shows the dependence of the roughness factor on pulse width (t_on_) and total t_on_ time or total deposition time (t_on_ × number of pulses). Each point represents the average of 64 electrodes; the standard deviation is depicted by semi-transparent layers below and above. The relative standard deviation throughout the parameter space is less than 10% (<4.5% for t_on_ = 48 s), which demonstrated the excellent reproducibility of the deposition procedure. The roughness factor (i.e., the ratio of active area to geometric area) is proportional to the total deposition or t_on_-on time as expected. The roughness factor can be increased to up to 150 µm^2^/µm^2^ (from the initial 1.6 µm^2^/µm^2^ on average before the deposition) without overgrowth of the recessed electrodes. Depositions at different pulse widths (t_on_) cannot be distinguished optically (using SEM). Reducing the relaxation time t_off_ down to 0.25 s did not visibly change the structures or the electrochemical response of the resulting deposited layer. However, the relaxation time may need to be increased for larger electrodes or closer spacing to allow for replenishing the depleted liquid volume around the deposition electrodes through diffusion in the liquid phase.

In Figure 4b the charge in the hydrogen desorption region, used for determining the roughness factor, is plotted against the maximum anodic pulse height of the pulse train during deposition (cf. asterisks in Figure 2). The plot reveals a clear linear dependence between pulse height and integrated charge (R^2^ = 0.985). The active area can thus be controlled and predicted during the deposition process. This may prove useful to fine tune the active area and impedance of the electrodes in order to achieve uniform electrode characteristics over a large array. 

### 3.2. Gold Deposition

Gold electrodes are widely used to attach biomolecules by using thiol chemistry. The strong affinity of the thiol groups for noble metal surfaces enables the formation of covalent bonds between the sulfur and gold atoms [14]. This biofunctionalization is a common approach used for nucleic-acid biosensors [3,25]. Hence, the voltage-pulse-based deposition protocol was evaluated for the deposition of gold on platinum electrodes. 

**Figure 5:**
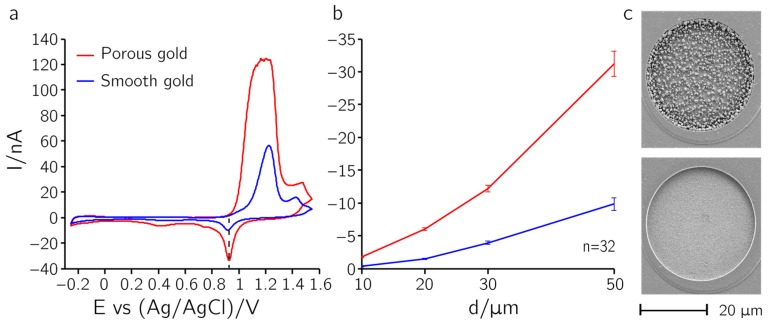
Gold deposition. Cyclic voltammogram (CV) of an electrode with deposited gold layer. (**a**) CV of 50-µm-diameter electrode modified with granular or smooth gold in 0.5 M H_2_SO_4_ in a voltage range between −0.25 V and 1.55 V vs. Ag/AgCl; the sweeping rate was 100 mV/s. A single sharp reduction current peak can be seen at ~0.9 V. Electrodes were completely covered so that no traces of platinum were visible. (**b**) Reduction current peak heights for different electrode diameters for smooth and granular gold layers; (**c**) scanning electron microscope (SEM) of a 30-µm-diameter electrode after deposition of granular gold (top) and smooth gold (bottom) layers.

The surface of the gold deposited on platinum electrodes can be rendered either smooth or granular by altering the deposition time (t_on_). Smooth gold depositions were achieved by using pulse durations (t_on_) of 0.25 s or longer, and granular gold depositions by using t_on_ times of 0.1 s. Figure 5a shows a CV in 0.5 M H_2_SO_4_ performed on 50-µm-diameter gold electrodes, using a sweep rate of 100 mV/s. One of the electrodes has been covered with granular gold and the other one with smooth gold. Complete coverage of the electrode can be deduced from the different potential window of gold with respect to that of platinum. The absence of contamination by other electroactive compounds is indicated by the presence of only the characteristic CV features of gold, including the single oxide reduction peak at approx. 0.9 V [26].

The total t_on_ time needed for complete coverage was 50 s for smooth and 150 s for granular gold. If the electrodes were not covered completely, the CV could not be performed in the specific range for gold electrodes (up to 1.6 V vs. Ag/AgCl), because the platinum surface then promoted the generation of gaseous oxygen above 1.2 V vs. Ag/AgCl, which led to excessive currents and overload of the potentiostat. The dependence of the peak height of the oxide reduction peak (marked with a dashed line in Figure 5a) on the electrode diameter is shown for smooth and porous layers in Figure 5b. The low standard deviations within the sets of values obtained from 32 electrodes that have been coated in parallel demonstrate the good reproducibility of the deposition process. Granular gold has an active surface area which is three times larger due to its dendritic nature. Further, the current peak height was proportional to the electrode area. The different surface textures—granular for the top graph and smooth for the lower graph—can clearly be seen in the SEM micrographs shown in Figure 5c. A close-up view of the granular gold layer is provided in Supplementary Figure S3.

### 3.3. Deposition of the Conducting Polymer PEDOT

PEDOT was chosen as an exemplary system to evaluate the proposed deposition scheme for conducting polymers. PEDOT with embedded enzymes has been used for devising biosensors [27,28], or enzymes have been grafted onto the conducting PEDOT polymer [29]. Furthermore, PEDOT has been used for neurotransmitter sensing [30] and as an anti-fouling layer [16].

**Figure 6:**
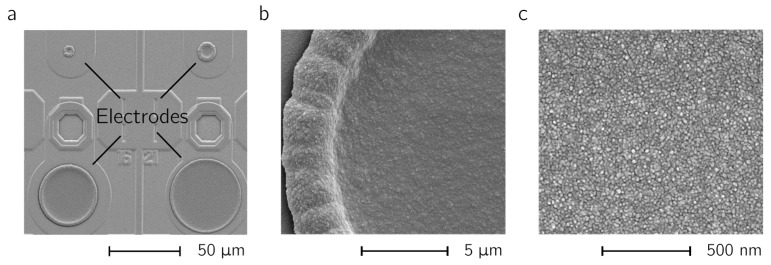
Deposited Poly(3,4-ethylenedioxythiophene) (PEDOT). (**a**) SEM micrographs of 5, 10, 40 and 50 µm diameter electrodes and (**b**,**c**) close-up views of a 25-µm-diameter electrode after PEDOT deposition.

**Figure 7:**
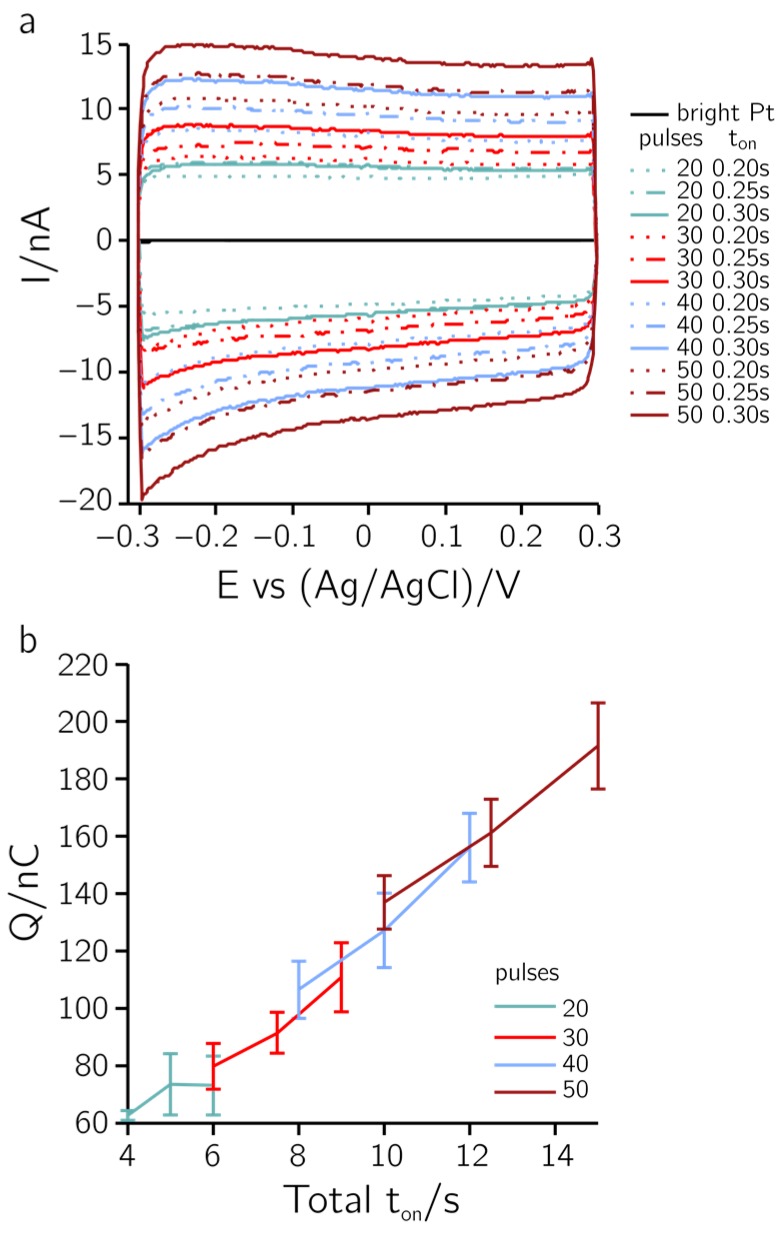
PEDOT. (**a**) CVs in phosphate-buffered saline (PBS) between −0.3 and 0.3 V vs. Ag/AgCl at 100 mV/s sweep rate, on 25-µm-diameter electrodes. The electrode materials included bright Pt and PEDOT deposited with a t_on_ of 0.2 s, 0.25 s, 0.3 s while applying 20, 30, 40, and 50 pulses. (**b**) Integrated total charge for different total-ton times. Colors code the number of pulses and correspond to (a). The integrated current over a CV of a bright Pt electrode is 200 pC.

Depositions on electrodes of different sizes are shown in the SEM micrographs of Figure 6a. A slight overgrowth at the rim of the electrodes was observed. The dense structure of the deposited layer can be seen in Figure 6b,c. Figure 7a shows CVs performed in PBS between –0.3 and 0.3 V vs. an Ag/AgCl reference electrode at a sweep rate of 100 mV/s. A large non-faradaic current can be observed as compared to a bare Pt electrode (black trace around the zero line in Figure 7a). In Figure 7b the integrated charge is plotted against the total t_on_ time. The results indicate that the total t_on_ time defines the electrode capacitance. The capacitance (C) can be estimated from the current values of the CV (C = current/sweep rate). For the values in Figure 7, the capacitances range between 50 and 150 nF (10–30 mF/cm^2^), which is approx. 1000 times larger than the characteristic capacitance measured using bright Pt electrodes (150 pF or 30 µF/cm^2^). In the SEM micrographs, there is no indication for an increase in the roughness of the deposited layers upon applying different total t_on_ times. Therefore, the active area cannot be attributed to an increased capacitance. PEDOT/PSS is known to exhibit a large pseudo-capacitance due to the immobilized anions (PSS^−^; [31]). Hence, the dependence of the pseudo-capacitance on the total t_on_ time observed in Figure 7 can be attributed to differences in the deposited layer thicknesses. 

### 3.4. Deposition of the Non-Conducting Polymer PPD

The voltage-pulse-based deposition procedure has also been applied for depositing a non-conducting polymer, poly(phenylenediamine (PPD). The non-conducting nature of PPD has been employed to prevent other electro-active molecules, such as dopamine or ascorbic acid, from being oxidized at the electrode surface and to thus avoid interference with the signal of interest [15]. Besides, PPD features perm-selective properties [23] and has been used as a matrix for embedding biosensitive elements [32].

**Figure 8:**
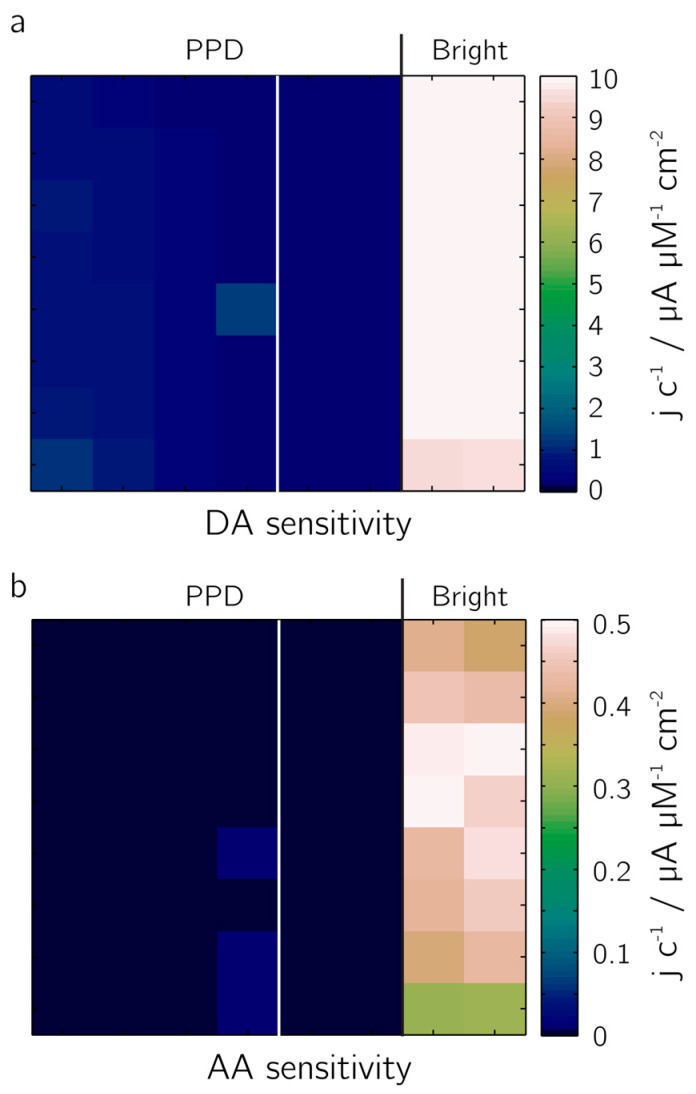
Non-conducting polymer poly(phenylenediamine (PPD). Sensitivity values (in current density per concentration) obtained from measurements of two blocks of 20-µm-diameter electrodes: the left block was completely covered with PPD; half of the right block was covered with PPD and half was left with bright Pt electrodes. Sensitivity was evaluated for (**a**) dopamine and (**b**) ascorbic acid. AA: ascorbic acid; DA: dopamine.

The rejection of the electro-active species dopamine, DA, (Figure 8a) and ascorbic acid, AA (Figure 8b) from the electrode surface has been demonstrated in an amperometric calibration series. Two 4 × 8 electrode blocks of 20-µm-diameter bright Pt electrodes were evaluated: the electrodes of the left block were completely covered with PPD, those of the right block were half covered with PPD, the other half was left bright Pt for comparison. Sensitivities were determined by measuring the currents upon addition of solutions of different concentrations of DA and AA (0–400 µM). The perm-selective nature of the coating became evident: the signal of the PPD-functionalized electrodes upon DA addition was by a factor of 24 lower, the signal upon addition of AA by a factor of 160, both in comparison to bright Pt electrodes. A variation of the deposition parameters did not yield a clear trend with regard to the suppression of DA or AA redox reactions at the electrodes. This may be due to self-limiting nature of the deposition of the non-conducting polymer whereby additional deposition cycles might not result in further deposition of the polymer, limiting the maximum layer thickness achievable by electodeposition-based growth.

## 4. Discussion

Galvanostatic electrodeposition allows for controlling and optimizing the deposition rate. When using a voltage scheme, the voltage can be tuned to facilitate specific reactions, instead of forcing reactions to happen at a certain rate, which is the case in applying current-based deposition schemes. Upon forcing electrochemical reactions to happen at a certain rate, the generated overpotentials may promote secondary reactions. These secondary reactions are undesirable, especially for functionalization of microelectrodes. The evolution of hydrogen, for example, can lead to holes in the deposited layers that then render the electrode functionalization useless. Instead, by defining the deposition potential, as has been done in the procedures presented here, secondary reactions could be obviated, and uniform, well-controlled layers were produced.

An advantage of current-based deposition schemes is the adaptation of the voltage during the deposition. This adaptation automatically accounts for a change of the electrode potential when a first layer of deposited material covers the electrode surface so that the deposition rate remains constant. The fact that this adaptation is missing in voltage mode seemed, however, not to be an issue in the depositions performed in this study. Upon entering diffusion-limited regimes or when applying self-limiting deposition processes, the use of current-based schemes will entail the risk of having high electrode potentials and, consequently, having unwanted secondary electrochemical reactions. This issue will not occur in voltage-based schemes, as the voltage is always precisely controlled, so that lower currents will result under voltage-controlled conditions. We found that voltage-controlled conditions were very well suited to achieve homogenous and reproducible deposition of a PPD layer, which features self-limiting characteristics.

Electrodeposition procedures are usually mass-transfer limited by diffusion. Pulsed-current or reverse-pulsed-current protocols are supposed to give better results, not in terms of deposition efficiency, but in uniformity of layer growth and tuning physical layer properties [17]. During the t_off_ time, the voltage-pulse-based deposition scheme presented here produces the same effects as pulse-reverse-current plating techniques: it counteracts surface charging, which may repel further ions, and it counteracts depletion of ions in areas, where deposition occurs [17]. The advantageous effects on diffusion lead to excellent uniformity of the deposited layers, which can be seen in the low standard deviations of the simultaneous parallel depositions of gold and platinum black.

The biggest advantage of the presented deposition scheme becomes evident upon applying it on large microelectrode arrays. In this case it is not necessarily deposition rate efficiency, but the flexibility in selecting the deposition area (which electrodes, how many electrodes, which electrode size), as well as a high yield and deposition uniformity, which are crucial. This flexibility of the deposition scheme is pivotal for depositions on electrodes of different sizes or diameters and for deposition on different numbers of electrodes, as has been shown.

The method can be adapted for other materials to be deposited according to the following procedure: (i) In order to determine E_off_, the open-circuit potential of the deposition solution has to be measured between an Ag/AgCl reference electrode and the electrode on which the deposition will be done; (ii) E_on_ should then be gradually increased (using the proper polarity for the deposition reaction) until deposition occurs; (iii) t_on_ should be kept small in order not to reach a diffusion limited regime (<1 s for our array dimensions); (iv) t_off_ should be set to a value at which no significant current can be detected anymore.

## 5. Conclusions

The voltage-pulse-based deposition scheme described in this study constitutes a robust method to achieve spatially controlled and uniform layer depositions of both metallic and polymeric films on large arrays of microelectrodes. In the case of Pt black, the active surface area of the individual electrodes could be increased in a controlled and reproducible manner while keeping the geometric electrodeposition area restricted to the exposed electrode surface. Applying the scheme for gold depositions, the morphology of the resulting gold layer could be varied by changing the pulse parameters. Voltage-pulse-based deposition further allows for fine-tuning the impedance of the commonly used conductive polymer PEDOT through variation of the layer thickness. Even self-limiting depositions (PPD) could be performed without the risk of obtaining large overpotentials. The method is particularly suitable for integrated microelectrode arrays, since the deposition waveforms can be readily generated by using basic electronic circuitry. The method is easily scalable, and the same parameters can be used to functionalize the surface of either single electrodes or large arrays of thousands of electrodes or varying sizes. Scale-up to larger electrode sizes, number or even to whole arrays is simply a matter of switching on the respective electrodes during deposition. Arbitrarily selectable subsets of large arrays of electrodes can be simultaneously functionalized for massive parallel sensing, or individual electrodes or selected subsets of electrodes can be flexibly functionalized for applications in multi-analyte measurements. Finally, the proposed method can be easily adapted to depositing other potential electrode materials by adapting the deposition voltage and pulse length parameters on a single or a few electrodes before then applying the method to larger numbers of electrodes or arrays.

## Figures and Tables

**Table 1:** Electrodeposition parameters for the different materials.

Material	t_on_	E_on_	t_off_	E_off_	Cycles
Pt black	0.1–0.8 s	0 V	0.4 s	0.68 V	100–250
Au smooth	0.25 s	–0.43 V	0.25 s	0.12 V	220–360
Au granular	0.1 s	–0.45 V	0.1 s	0.12 V	2000
PEDOT	0.2–0.3 s	1.05 V	0.5 s	0.275 V	20–50
PPD	0.25 s	0.5–0.9 V	0.5 s	0.05 V	60–500

PEDOT: poly(ethylenedioxythiophene); PPD: poly(phenylenediamine).

## Supplementary Materials

The following are available online at http://www.mdpi.com/1424-8220/17/1/22/s1, Figure S1: Fully integrated electrochemical complemenary metal-oxide-semiconductor (CMOS) system: (a) packaged CMOS chip for electrochemical measurements. On the left are the gold contacts for data communication and power supply. In the middle is the opening to the array of 32 × 32 Pt microelectrodes. (b) The assembled system with a reservoir for the liquids. A standard Ag/AgCl reference electrode was immersed in the electrolyte. Figure S2: Cyclic voltammograms (CV) of H_2_SO_4_ on Pt black: Pt black characterization via a CV measured in a 0.5 M H_2_SO_4_ solution at a sweep rate of 100 mV/s. The CV was controlled and recorded by the fully integrated CMOS chip. The hatched area was integrated to determine the active surface area and the roughness factor displayed in Figure 4a,b. Figure S3: Granular Au: Close-up view of the surface of the granular gold in Figure 5c.
