# Supplementary material for: Robust Functionalization of Large Microelectrode Arrays by Using Pulsed Potentiostatic Deposition
Source: Sensors (Basel). 2016 Dec 23;17(1):22. doi: 10.3390/s17010022 (PMC5298595; doi:10.3390/s17010022)
Supplement: Supplementary file 1 [file sensors-17-00022-s001.pdf]

Supplementary Materials: Robust Functionalization of Large Microelectrode Arrays by Using Pulsed Potentiostatic Deposition

Joerg Rothe, Olivier Frey, Rajtarun Madangopal, Jenna Rickus and Andreas Hierlemann

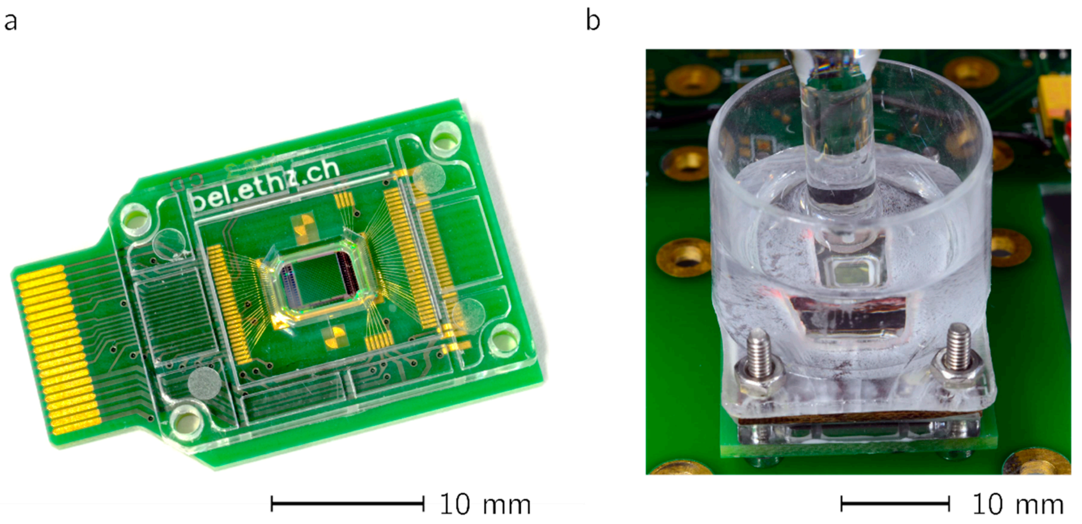

Figure S1. Fully integrated electrochemical complemenary metal-oxide-semiconductor (CMOS) system: (**a**) packaged CMOS chip for electrochemical measurements. On the left are the gold contacts for data communication and power supply. In the middle is the opening to the array of 32 × 32 Pt microelectrodes. (**b**) The assembled system with a reservoir for the liquids. A standard Ag/AgCl reference electrode was immersed in the electrolyte.

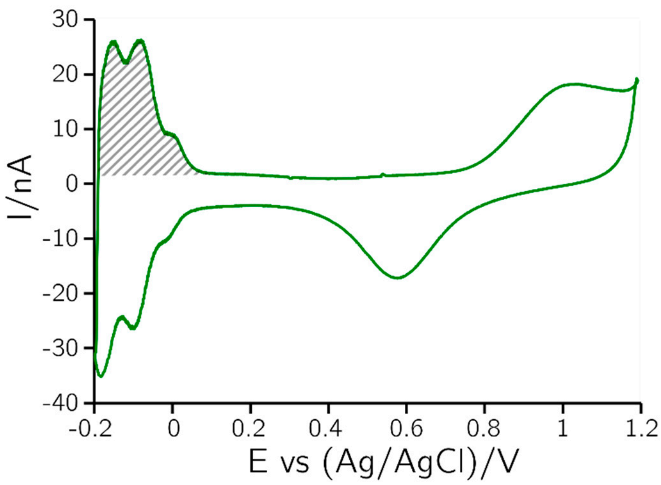

Figure S2. Cyclic voltammograms (CV) of H_2SO_4 on Pt black: Pt black characterization via a CV measured in a 0.5 M H_2SO_4 solution at a sweep rate of 100 mV/s. The CV was controlled and recorded by the fully integrated CMOS chip. The hatched area was integrated to determine the active surface area and the roughness factor displayed in Figure 4a,b.

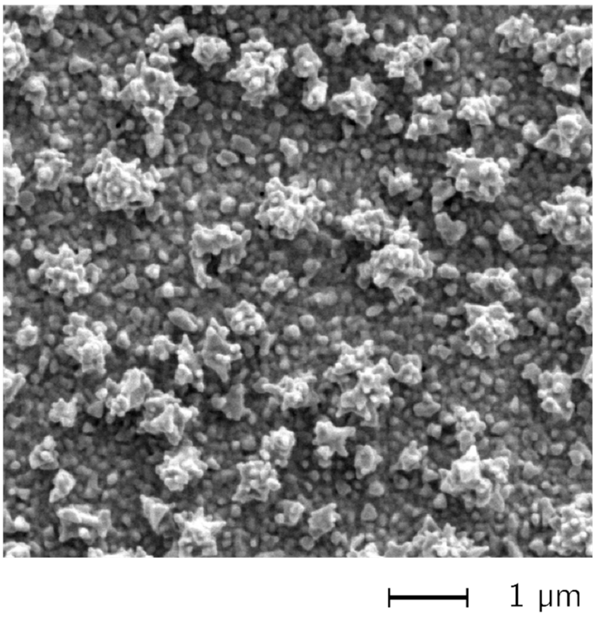

Figure S3. Granular Gold: Close-up view of the surface of the granular gold in Figure 5c.
